# Towards Noninvasive Detection of Oesophageal Varices

**DOI:** 10.1155/2012/343591

**Published:** 2012-03-14

**Authors:** Kara Rye, Robert Scott, Gerri Mortimore, Adam Lawson, Andrew Austin, Jan Freeman

**Affiliations:** Liver Unit, Royal Derby Hospital, Uttoxeter Road, Derby DE22 3NE, UK

## Abstract

Current guidelines recommend that all cirrhotic patients should undergo screening endoscopy at diagnosis to identify patients with varices at high risk of bleeding who will benefit from primary prophylaxis. This approach places a heavy burden upon endoscopy units and the repeated testing over time may have a detrimental effect on patient compliance. Noninvasive identification of patients at highest risk for oesophageal varices would limit investigation to those most likely to benefit. Upper GI endoscopy is deemed to be the gold standard against which all other tests are compared, but is not without its limitations. Multiple studies have been performed assessing clinical signs and variables relating to liver function, variables relating to liver fibrosis, and also to portal hypertension and hypersplenism. Whilst some tests are clearly preferable to patients, none appear to be as accurate as upper GI endoscopy in the diagnosis of oesophageal varices. The search for noninvasive tests continues.

## 1. Introduction

Cirrhosis is the end stage of every chronic liver disease, resulting in formation of fibrous tissue, disorganization of liver architecture, and nodule formation, which interferes with liver function and results in portal hypertension. Portal hypertension is associated with development of a hyperdynamic circulation and complications such as ascites, hepatic encephalopathy, and oesophago-gastric varices. Patients with cirrhosis and gastro-oesophageal varices have a hepatic venous pressure gradient during haemodynamic catheterization of at least 10–12 mmHg [1]. Oesophageal varices are present at diagnosis in approximately 50% of cirrhotic patients, being more common in Child-Pugh class C patients compared to Child-Pugh class A patients (85% versus 40%) [1, 2]. De novo formation of varices occurs at a rate of 5% per year, with a higher incidence in patients continuing to consume alcohol or with worsening liver function [2]. Once varices form, they enlarge from small to large at a rate of 5–12% per year [2] and bleed at a rate of 5–15% per year. The greatest bleeding risk is seen in large varices classified as being >5 mm diameter and is also influenced by liver disease severity as assessed by Child-Pugh score, and by the presence of red wale markings on varices at endoscopy. Therefore, these factors should also be taken into consideration to classify “high-risk varices” [3].

Reports from the 1940's to the 1980's demonstrate poor outcomes from variceal bleeding with mortality rates between 30–60% [4–6], but studies suggest that the outcomes have improved over the last few decades [7–9]. This is demonstrated in a study by Carbonell et al. [10], who showed that between 1980–2000, the inhospital mortality from variceal bleeding decreased from 42.6% to 14.5% and was associated with decreased rebleeding and rates of bacterial infection. 

Although mortality from a bleeding episode has decreased with improved endoscopic and radiological techniques together with new pharmacologic therapies, a 20–30% mortality [11] means that bleeding from oesophageal varices remains of significant clinical importance. Early diagnosis of varices before the first bleed is essential as studies of primary prophylaxis clearly show that the risk of variceal haemorrhage can be reduced by 50% to about 15% for large oesophageal varices [12]. Current guidelines, therefore, recommend that all cirrhotic patients should be screened for varices at diagnosis, with followup every 2-3 years for patients without varices (depending upon liver disease severity) and 1-2 years for patients with small varices, to assess for enlargement of varices and need for prophylactic treatment [13]. Upper GI endoscopy remains the gold standard for screening, but this test is not without its own limitations. There is conflicting evidence with regard to the interobserver agreement for endoscopic diagnosis of variceal presence, grade, or presence of red signs [14–16]. Cales et al. found in 100 cirrhotic patients that the interobserver agreement between four independent observers for the size of oesophageal varices and presence of red signs was good with kappa values of 0.59 and 0.60, respectively. However, Bendtsen et al. found considerable variation in the interobserver agreement on the diagnosis and grading of oesophageal varices between 22 endoscopists with a large variation in kappa values. The current guidelines cause a significant burden and cost to endoscopy units and necessitate patients having repeated unpleasant procedures even when up to 50% may still not have developed oesophageal varices 10 years after the initial diagnosis [2].

If it were possible to predict oesophageal varices by noninvasive means this would restrict testing to the population deemed to be at most risk and reduce the number of endoscopies required. Such a screening test should be simple, quick, reproducible, and cost effective. The utility of current noninvasive tests to predict oesophageal varices will be reviewed in this paper.

## 2. Current Perspectives: Possible Approachesto Noninvasive Diagnosis of Oesophageal Varices

### 2.1. Physical Examination and Laboratory Parameters

Several studies have examined the usefulness of different clinical and laboratory parameters as predictors of the presence or size of oesophageal varices. These are discussed below.

#### 2.1.1. Physical Signs and Variables Related to Liver Function

A number of clinical signs and other laboratory markers have been identified either alone or in combination as factors predicting the presence of oesophageal varices. These include the presence of spider naevi, splenomegaly or ascites, Child-Pugh classification, serum albumin, and prothrombin time.

Spider naevi, a low-albumin and low-platelet count were shown to be independent risk factors for the presence of varices in a study by Garcia-Tsao et al. [17]. In a further study by Berzigotti et al. [18], spider naevi, ALT, and albumin were found to predict oesophageal varices with the best cutoff giving a sensitivity of 93%, specificity of 37%, and correctly classifying 72% of patients. Similarly, spider naevi have been found to be predictive of large oesophageal varices with a diagnostic accuracy of 72% when using the variables platelet count, prothrombin index, and spider naevi [19]. Chalasani et al. [20] found that splenomegaly detected on clinical examination was an independent risk factor for the presence of large varices. Zaman et al. [21] demonstrated that cirrhotic patients in Child-Pugh classes B or C were almost 3 times as likely to have oesophageal varices or large oesophageal varices as compared to patients in Child-Pugh class A. 

The Baveno IV International Consensus Workshop on methodology of diagnosis and treatment concluded that no study reached a high enough level of significance to warrant the widespread use of such noninvasive markers of oesophageal varices [13].

#### 2.1.2. Variables Related to Liver Fibrosis

Chronic liver injury and inflammation leads to fibrosis and ultimately cirrhosis, through the deposition of extracellular matrix (ECM) complexes. The collagen fibrils of the complex undergo secondary processing, becoming cross-linked, which confers resistance to degradative enzymes and irreversibility [22]. Normally, deposition of the ECM is a dynamic, reversible process with removal of ECM mediated by several specific matrix metalloproteinases (MMPs), which in turn are regulated by soluble inhibitors termed TIMPs (tissue inhibitor of metalloproteinase). A number of serum markers for ECM deposition and removal have been evaluated as candidate markers for liver fibrosis, and a small number of studies have evaluated their usefulness in predicting oesophageal varices. Potential markers examined to date include the glycoproteins, hyaluronic acid and laminin, and members of the collagen family including procollagen III and type IV collagen. Conflicting results have been demonstrated. Galal et al. [23] assessed the ability of serum hyaluronic acid to predict medium-to-large oesophageal varices and showed the sensitivity, specificity, positive and negative predictive values, and diagnostic accuracy at a cutoff of 207 *μ*g/L to be 94%, 77.8%, 88.7%, 87.5%, and 88.3%, respectively. Körner et al. [24] showed no association between concentrations of hyaluronic acid or laminin and grade of oesophageal varices, and a further study by Bahr et al. [25] confirmed the lack of association of serum laminin to size of oesophageal varices. 

Similar conflict is seen when examining the evidence with regard to the role of the collagens. In the first of only 2 studies in this area, the aminoterminal propeptide of type III procollagen was shown to have a weak correlation to the degree of oesophageal varices [26]. The second study by Mamori et al. [27] included 44 patients with alcoholic liver disease and demonstrated a significant difference in serum type IV collagen levels between patients with and without varices (712.3 versus 404.3 ng/mL, *P* < 0.001), giving an AUROC of 0.78 for predicting the presence of oesophageal varices.

None of the aforementioned markers could currently be utilised to predict oesophageal varices in portal hypertension; in view of this several different biomarkers have been combined with the aim of improving their diagnostic ability. FibroTest is a composite score generated by combining the results of five serum blood tests (alpha-2-macroglobulin, apolipoprotein A1, haptoglobin, *γ*-glutamyltranspeptidase, and bilirubin and alanine) corrected for the age and gender of the patient. Results have shown high predictive values for significant fibrosis in patients with chronic hepatitis C, chronic hepatitis B, fatty liver disease, and chronic alcoholic liver disease [28–31]. A single study has assessed the predictive value of fibroTest in the diagnosis of large oesophageal varices in 99 cirrhotic patients [32]. Significant differences in FibroTest value (0.89 versus 0.82), platelet count (110 versus 150), and prothrombin time (50 versus 66%) were seen between patients with and without large oesophageal varices. FibroTest had the highest discriminative power of all the variables with an AUROC curve of 0.77. Using a cutoff of 0.80, this gave a sensitivity of 92%, specificity 21%, PPV 33%, and NPV 86%. A fibroTest score < 0.75 was found to be associated with the absence of large oesophageal varices with a NPV of 100%. The limitations to the study are that it was a retrospective study with significant population bias and has not been reproduced in a prospective study of compensated cirrhotics. FibroTest is not readily available to most clinicians, which limit its utility as a screening test.

#### 2.1.3. Variables Related to Portal Hypertension and Hypersplenism

Thrombocytopenia may occur in portal hypertension-induced splenomegaly, in part due to platelet sequestration, and a large number of studies have been performed assessing the relationship between platelet count and oesophageal varices [17, 19–21]. A low-platelet count is regularly identified as predictive of oesophageal varices and large oesophageal varices, but there is a wide variation in the cut-off level of platelets used, ranging from 68,000 to 160,000 with sensitivities ranging from 71–90% and specificities from 36–73%. Bias is likely to account for much of this variation, with the majority of studies being retrospective in nature, having heterogeneous cohorts of patients resulting in both selection and spectrum bias. 

A longitudinal study by Qamar et al. [33] of 213 patients, with compensated cirrhosis with portal hypertension but without varices, demonstrated that the median platelet count at the time of occurrence of varices was 91,000. However, no platelet count could be identified that accurately predicted the presence of oesophageal varices (AUROC curve 0.63), and they, therefore, concluded that platelet count is an inadequate noninvasive marker for prediction of the presence of oesophageal varices. In an attempt to improve the predictive value of the platelet count, it has been combined with other variables, and the results of these studies are discussed below. 

Oesophageal collaterals develop as a consequence of portal hypertension, being formed by vascular remodelling and angiogenesis. Key molecules thought to be involved in this include nitric oxide and vascular endothelial growth factor (VEGF). A single study of 85 cirrhotic patients examined the predictive capability of serum nitrate levels to detect oesophageal varices [34]. Significant differences in serum nitrate levels were found between patients with large oesophageal varices compared to patients without oesophageal varices (*P* < 0.01). The best cut-off level for prediction of oesophageal varices was 38 *μ*mol/L, giving a sensitivity 86.5%, specificity 83.3%, PPV 95%, and NPV 62.5%. Animal studies suggest that the formation of oesophageal varices results not only from opening up of preexisting collateral vessels but also as a result of angiogenesis which may in part be mediated by VEGF. Use of VEGF as a noninvasive biomarker has only been investigated in a single study, and no correlation between VEGF levels and grade of oesophageal varices was detected [35].

The development of portosystemic collaterals and the resultant shunting is responsible for the complication hepatic encephalopathy, in which ammonia plays a role. One study has examined the role of blood ammonia concentrations in the noninvasive detection of oesophageal varices [36]. In this study of 153 cirrhotic patients, a significant correlation was demonstrated between oesophageal variceal grade and venous ammonia levels (*r* = 0.43, *P* < 0.001). The AUROC curve for predicting the presence of oesophageal varices was 0.78, and using a cut-off of 42 *μ*M/L this gave a sensitivity of 92% and a specificity of 60%.

Therefore, variables associated with portal hypertension and hypersplenism are not accurate enough to be used as noninvasive markers of oesophageal varices.

#### 2.1.4. Predictive Scores

**(1) Platelet Count/Spleen Diameter Ratio**

This ratio is calculated by dividing the platelet number/mm^³^ by the maximum spleen bipolar diameter in mm as estimated by abdominal ultrasound. There have now been a number of studies assessing this. The first by Giannini et al. in 2003, reported the platelet count/spleen diameter ratio to be the only independent variable associated with presence of OV on multivariate analysis and identified a cut-off value of 909, giving a PPV of 96% and NPV of 100% [37]. The second part of the study confirmed the reproducibility of this cut-off level with a PPV of 74% and NPV of 100% in compensated cirrhotic patients. The same group then followed up 68 patients without OV with repeat endoscopy and calculation of the platelet/spleen diameter ratio. At followup, patients with a platelet count/spleen diameter ratio < 909 had 100% NPV and 84% PPV, and they concluded that the platelet count spleen diameter ratio was effective in ruling out the presence of OV when cirrhotic patients were followed longitudinally. Subsequently, a multicentre, international validation study using the 909 ratio was performed in 218 patients [38]. The test performed less well than in the original study with a PPV of 76.6% and a NPV of 87.0%. This has been a consistent feature in all studies subsequently performed which vary from being retrospective or prospective in nature and utilise different cut off points [39–43]. Therefore, despite promising early results the platelet count/spleen diameter ratio is not a reliable tool to screen for oesophageal varices.

**(2) Platelet Count and Child-Pugh Class**

In 2007, Burton et al. published the validation of a model for predicting size and presence of varices based upon platelet count and Child-Pugh class [44]. The first model aimed to detect large varices in Child-Pugh A patients with a platelet count <80 and had a sensitivity of 58%, specificity 79%, PPV 30%, and NPV 92%. The second model aimed to identifying any varices in Child B/C patients with a platelet count <90 and had a sensitivity of 60%, specificity of 59%, PPV 80%, and NPV 34%. Once again, the performance of these models would not reliably predict the presence of oesophageal varices.

**(3) AST/ALT Ratio**

The AST/ALT ratio has been used to predict cirrhosis, and by natural extension studies have been performed to assess its usefulness in predicting oesophageal varices. In a retrospective study [45], significantly higher AST/ALT ratios were seen in patients with varices compared to those without (ratio: 1.8 versus 1.0, *P* < 0.0001). A further prospective study [46] found an AST/ALT ratio > 1.12 to be significantly associated with the presence of varices at initial endoscopy (OR 3.9, *P* = 0.02 95% CI 1.3–11.8). This cutoff gave a sensitivity of 47.8%, specificity of 87%, PPV 42.3%, and NPV 89.2%, and an AUROC of 0.69. A further study using a different cut-off of ≥1.0 demonstrated a sensitivity of 68%, specificity of 89%, PPV 77%, and NPV 83%, with an AUROC 0.83 (0.72–0.94) for predicting the presence of oesophageal varices [47]. For the prediction of large oesophageal varices, this gave a sensitivity 68%, specificity 77%, PPV 41%, and NPV 92%, and AUROC 0.79 (0.64–0.94). Overall, the AST/ALT ratio correctly classified 81% patients for the detection of varices and 76% of those with large varices. Therefore these studies, which include patients with different aetiologies of liver disease and used different cutoffs for the AST/ALT ratio cannot confidently predict the presence of oesophageal varices in clinical practice to avoid screening all cirrhotic patients with endoscopy.

**(4) Right Lobe Liver Albumin Ratio**

This ratio is calculated by dividing the right liver lobe diameter (as assessed by abdominal ultrasound and measured in millimetres) by the serum albumin concentration (g/L). This has been assessed in a single study of 94 cirrhotic patients [48]. Right liver lobe/albumin ratio correlated with presence and size of oesophageal varices (*r* = 0.488, *P* < 0.01; *r* = 0.481, *P* < 0.01, respectively). For a cut-off value of 4.425 this gave a sensitivity of 83.1% and specificity 73.9% and thus once again cannot be used as a reliable screening test.

### 2.2. Transient Elastography

#### 2.2.1. Liver Stiffness

Transient elastography (TE, FibroScan, Echosens, France) is a noninvasive technique developed to assess hepatic fibrosis in patients with chronic liver diseases. Fibrosis causes an increase in liver stiffness, and measurement of this forms the basis of TE, which is painless, rapid, and easy to perform. Studies suggest that TE is highly reproducible and reliable with very high interobserver and intraobserver agreement overall but that patient related and liver disease related factors may have a negative effect on the reproducibility of this technique [49]. A wide range of liver stiffness values have been reported ranging from 2.5 to 75 kPa, being influenced by gender, body mass index, disease aetiology, and presence of necroinflammatory change [50–53]. As a rough guide, normal TE values are considered to be 3.8–8 kPa in men and 3.3–7.8 kPa in women, significant fibrosis (Metavir fibrosis stage ≥ 2) 7-8 kPa and cirrhosis 13–17 kPa.

A number of studies have been performed examining the relationship of liver stiffness to size and presence of oesophageal varices, and these results are summarised in Table 1 [47, 54–57]. These studies demonstrate a significant correlation between liver stiffness measurements and the presence of oesophageal varices but are divided with regard to the relationship of liver stiffness to variceal size. 

For the diagnosis of variceal presence, AUROC curves varied from 0.76–0.85, with a sensitivities of 84–95%, specificities of 43–78%, PPV 57–89%, and NPV 66–91% using cutoffs between 13.9–21.5 kPa. For the diagnosis of large oesophageal varices, AUROC varied from 0.76–0.87, with sensitivities of 77–91%, specificities of 60–85%, PPV 48–56% and NPV 94-95% using cut-offs between 19–30.5 kPa. The other limitations of the study relate to inclusion of patients with liver disease of different aetiologies and of different severity, according to Child-Pugh class.

The study by Castera et al. best represents the cohort of patients in whom noninvasive screening for varices is needed [47]. All 70 patients were Child-Pugh class A and had cirrhosis secondary to hepatitis C. They demonstrated that LSM values increased with the grade of OV (*P* < 0.001). The AUROC for presence of OV was 0.84 and 0.87 for large OV. A cutoff of 21.5 kPa predicted the presence of OV with a sensitivity of 76%, specificity 78%, PPV 68%, and NPV 84% and correctly classified 73% of patients. At a cutoff of 30.5 kPa, the presence of large OV was predicted with a sensitivity 77%, specificity 85%, PPV 56%, and NPV 94%, and correctly classified 79% of patients.

Therefore, the predictive performance of liver stiffness measurement is poor for the diagnosis of OV with low specificity and PPV, particularly with regard to large OV. However, it may be useful as a screening test to identify patients in whom variceal screening is not required, but at present cannot be advocated as a surrogate for gastroscopy.

#### 2.2.2. Spleen Stiffness

Transient elastography has also been used to determine spleen stiffness, using the hypothesis that splenomegaly resulting from portal hypertension causes changes in the spleen's density. In a study of 191 patients (135 cirrhotic) recently published, it was demonstrated that spleen stiffness was significantly higher in cirrhotics than noncirrhotics and in patients with oesophageal varices compared to those without [58]. 52.5 kPa was determined to be the best cutoff giving an AUROC curve of 0.74. They found a better diagnostic accuracy, of 89.95%, in predicting the presence but not the grade of oesophageal varices when liver and spleen stiffness were used together. 

MR Elastographic spleen stiffness has also been assessed in a small study of 17 compensated cirrhotics. All of the 7 patients with oesophageal varices had a mean spleen stiffness of >10.5 kPa [59]. Further larger studies are needed to investigate the diagnostic accuracy of MR Elastographic spleen stiffness for noninvasive prediction of oesophageal varices. 

### 2.3. Other Imaging Modalities

#### 2.3.1. Ultrasound

Doppler ultrasonography (US) imaging provides a real-time, inexpensive, and repeatable examination of the portal system and allows estimation of both arterial and venous flow. It is considered the first-line imaging technique in patients with cirrhosis. Portal vein diameter, portal blood velocity and congestion index, spleen size, flow pattern in the hepatic veins, and the presence of abdominal portosystemic collaterals are all US parameters previously thought to have with prognostic significance but all with poor sensitivity and specificity [60]. One large study proposed prothrombin activity of less than 70%, portal vein diameter greater than 13 mm, and platelet count < 100 × 10^9^ as noninvasive predictive tools to discriminate cirrhotic patients with and without oesophageal varices (OV) [61]. The ROC curve constructed from all possible combinations of these dichotomous variables initially looked promising with an area under the curve (AUC) value of 0.80. To assess the validity of this tool, the investigators used a matched second cohort where the positive predictive value was found to be significantly reduced [62]. A further validity study was repeated in another centre with a similarly poor sensitivity and specificity [63]. Thus, US has limited specificity and cannot replace endoscopy as a screening tool for large oesophageal varices [61, 63].

#### 2.3.2. CT

Three recent studies suggest that multidetector CT is comparable to upper endoscopy in detecting small and large varices [64–66]. Only two of these studies were carried out prospectively [64, 65], and only one included a cost analysis [64]. In one of the previous studies, virtual oesophagography could be carried out using the CT scans, but this procedure requires time-consuming and invasive intubation of the oesophagus with a catheter for air insufflation [66]. CT was found to have approximately 90% sensitivity in the identification of oesophageal varices determined to be large on endoscopy, but only about 50% specificity. The sensitivity of CT detecting gastric varices was 87%. In addition, a significant number of gastric varices, perioesophageal varices and extraluminal pathology were identified by CT that were not identified by endoscopy. Use of CT as the initial screening modality for the detection of varices was significantly cost effective compared to endoscopy irrespective of the prevalence of large varices [64]. Patients overwhelmingly preferred CT over endoscopy in all three studies. One of the major limitations identified in all studies was the differing rates of interobserver agreement in variceal size of both modalities, with only one study finding agreement between radiologists being higher than between endoscopists [64]. How reproducible this model could, therefore, remain unproven. There are also major concerns over the risk of cumulative radiation exposure in prolonged screening programmes [67].

### 2.4. Capsule Endoscopy

New capsule endoscopy devices have been developed, specifically for use in the oesophagus, acquiring images from both ends of the device. Several studies have been performed, assessing the ability of these capsule endoscopy devices to detect any varices and identify large varices requiring primary prophylaxis [68–73]. Conventional OGD was used as the gold standard.

With regards to the detection of varices, sensitivity varied between 68–100%, and specificity 86–100% [70–72, 74]. In the largest study performed to date, 288 patients were recruited in a multicentre trial [68]. Conventional OGD identified OV in 180 patients (62.5%) and capsule endoscopy identified OV in 152 of these, giving a difference in diagnosing OV of 15.6% in favour of OGD. In 13 cases (14.5%), varices were identified by capsule but not confirmed by OGD. Overall agreement for detection of varices was 85.8%, with a sensitivity of 84%, specificity 88%, positive likelihood ratio 7.0, and negative likelihood ratio 0.18. With regard to the grading of varices, there was complete agreement on the grade in 79%. In differentiating between varices requiring treatment or not, the sensitivity, specificity, PPV, and NPV for capsule endoscopy were 78%, 96%, 87%, and 92%, respectively. Overall agreement on treatment decisions based on variceal size was 91% (kappa = 0.77). Other studies have correctly identified patients requiring primary prophylaxis in 74–100% of patients [69, 71, 72, 74]. 2 meta-analyses produced similar results with pooled sensitivities of 83% and 83.8% and pooled specificities of 85% and 80.5%, respectively for the diagnosis of oesophageal varices [75, 76].

Capsule endoscopy is reported to be feasible in 94–99% of patients with the main reasons for failure being because patients were unable to swallow the capsule or due to technical problems with the recording or function of the capsule. Adverse events have been reported in 0–1.4% of cases, including episodes of capsule retention necessitating removal. Tolerability of the capsule is found to be better than conventional OGD, with better preprocedure perception and postprocedure satisfaction. 26–83% patients prefer capsule endoscopy over conventional OGD in the studies performed to date [68–70, 72–74, 77].

With regard to cost-effectiveness, 2 studies have been performed, the first concluding that both screening methods are equivalent, the second that screening with capsule endoscopy followed by beta-blocker therapy may be cost-effective compared to OGD followed by beta-blocker therapy but is highly sensitive to local costs [78, 79].

Therefore in summary, capsule endoscopy is feasible in the majority of patients and with regard to patient preference, capsule endoscopy appears to be preferable to conventional endoscopy and may improve compliance with screening programmes, although this remains to be determined. The jury is still out with regard to cost but when it comes to performance, conventional OGD remains the gold standard.

## 3. The Future Approach to Noninvasive Detection of Oesophageal Varices

Cirrhosis and portal hypertension are characterized by the development of a hyperdynamic circulation with elevated cardiac output and stroke volume and reduced systemic vascular resistance [80]. These haemodynamic variables are independently associated with portal pressure and size of oesophageal varices [81–84]. Measurement is traditionally invasive, the thermodilution technique requiring introduction of a catheter into the pulmonary artery. A noninvasive method for assessing systemic haemodynamics may allow noninvasive detection of oesophageal varices. New techniques are now available that measure systemic haemodynamics noninvasively. The Finometer (Finapres Medical Systems, Amsterdam, The Netherlands) is a noninvasive device that allows continuous beat-to-beat blood pressure and haemodynamic monitoring over a number of hours [85]. We have demonstrated the presence of the hyperdynamic circulation using this technique and shown significant differences in cardiac output and systemic vascular resistance according to the size of oesophageal varices. We have also shown significant correlation of these haemodynamic variables to the 1-year probability of variceal bleeding. Data as yet unpublished examining the predictive ability of noninvasive parameters has shown promising initial results, with an AUROC curve of 0.86 for cardiac output and 0.77 for peripheral vascular resistance for the diagnosis of large oesophageal varices. Optimal cutoffs for these haemodynamic parameters remain to be defined. Considering a cutoff of 7.06 L/min for cardiac output, this gave a sensitivity of 91% and a negative predictive value of 93%, maintaining a diagnostic accuracy of 86%. Using a cutoff of 0.99 MU for peripheral vascular resistance gave a sensitivity of 91% and negative predictive value of 91%. These initial results require further investigation.

Proteomics is the large-scale study of proteins, particularly their structure and function and interactions in a biological system. Proteomics does not require prior knowledge of the proteins present and, therefore, is ideal to screen for the best biomarkers of disease. Promising results have been seen in patients with liver cirrhosis to search for markers of hepatic fibrosis [86–88] and has been demonstrated to be more accurate than fibroTest. The optimal biomarker needs to be able to predict clinically significant endpoints as well as liver histology, and so further research is needed to know whether proteomics will ever be useful in the noninvasive diagnosis of oesophageal varices.

The major significant endpoint with regard to varices is that of bleeding. The evidence shows that infection and variceal bleeding are related [89]. In experimental cirrhosis, bacterial products increase portal pressure by activating macrophages and releasing vasoconstrictive prostaglandins [90–92]. Soluble CD163 in serum is a new specific marker of macrophage activation. A recent study demonstrated that sCD163 is increased in cirrhosis, levels correlating with portal pressure, but that levels do not drop following reduction of portal pressure after transjugular intrahepatic portosystemic shunt [93]. Therefore, chronic activation of these cells may play a role in establishing and maintaining portal hypertension. Further work is needed to assess their potential not only as a noninvasive marker of oesophageal varices but of varices with the highest bleeding risk.

## 4. Conclusions

In conclusion, based on all the available evidence to date, upper GI endoscopy remains the gold standard for the diagnosis of oesophageal varices in cirrhotic patients despite its own limitations. Clinical, biochemical, and radiological parameters currently are not accurate enough to avoid screening endoscopy, due to the risks associated with missing patients with large oesophageal varices. A screening test must be simple and inexpensive, and therefore current promising tools such as CT scanning or capsule endoscopy which are highly acceptable to patients may not prove to be cost-effective or suitable for repeated measurement. Assessment of systemic haemodynamics and other serum markers may hold promise for the future, and more studies are needed to better understand and identify high risk groups, which may in time be facilitated by proteomic approaches.

## Figures and Tables

**Table 1:** Summary of diagnostic accuracy of LSM for the detection of oesophageal varices (OV) or large varices (LOV). TE: transient elastography.

Diagnostic performance of TE for the diagnosis of OV by Author	[54]	[55]	[56]	[57]	[47]
Number of pts	165	61 (47 cirrhotic)	150 (89 cirrhotic)	112	298 (70 cirrhotic)
Aetiology	Mixed (HCV predominant)	HCV	Mixed	Mixed	HCV
Prevalence OV	45%	64%	72%		36%
Proposed cutoffs forpresence of OV/LOV	OV 13.1 LOV 19	17.6	OV 21.1 LOV 29.3	19.7	OV 21.5 LOV 30.5
Sensitivity (%)	95/91	90	84/81	87	76/77
Specificity (%)	43/60	43	71/61	70	78/85
PPV (%)	57/48	77		89	68/56
NPV (%)	91/95	66		66	84/94
AUROC	0.84/0.83	0.76	0.85/0.76	0.818	0.84/0.87

## References

[1] Garcia-Tsao G, Sanyal AJ, Grace ND, Carey W (2007). Prevention and management of Gastro-oesophageal varices and variceal haemorrhage in cirrhosis. AASLD Practice Guideline. *Hepatology*.

[2] Merli M, Nicolini G, Angeloni S (2003). Incidence and natural history of small esophageal varices in cirrhotic patients. *Journal of Hepatology*.

[3] The North Italian Endoscopic Club for the Study and Treatment of Esophageal Varices (1988). Prediction of the first variceal haemorrhage in patients with cirrhosis of the liver and esophageal varices. A prospective multi-center study. *The New England Journal of Medicine*.

[4] Graham DY, Smith JL (1981). The course of patients after variceal hemorrhage. *Gastroenterology*.

[5] Nachlas MM, O’Neil JE, Campbell AJ (1955). The life history of patients with cirrhosis of the liver and bleeding esophageal varices. *Annals of Surgery*.

[6] Cortez Pinto H, Abrantes A, Esteves AV, Almeida H, Pinto Correia J (1989). Long-term prognosis of patients with cirrhosis of the liver and upper gastrointestinal bleeding. *American Journal of Gastroenterology*.

[7] Jamal MM, Samarasena JB, Hashemzadeh M (2008). Decreasing in-hospital mortality for oesophageal variceal hemorrhage in the USA. *European Journal of Gastroenterology and Hepatology*.

[8] Chalasani N, Kahi C, Francois F (2003). Improved patient survival after acute variceal bleeding: a multicenter, cohort study. *American Journal of Gastroenterology*.

[9] McCormick PA, O’Keefe C (2001). Improving prognosis following a first variceal haemorrhage over four decades. *Gut*.

[10] Carbonell N, Pauwels A, Serfaty L, Fourdan O, Lévy VG, Poupon R (2004). Improved survival after variceal bleeding in patients with cirrhosis over the past two decades. *Hepatology*.

[11] Pagliaro L, D’Amico G, Pasta L, de Franchis R Efficacy and efficiency of treatments in portal hypertension.

[12] D’Amico G, Pagliaro L, Bosch J, Patch D (1999). Pharmacological treatment of portal hypertension: an evidence-based approach. *Seminars in Liver Disease*.

[13] De Franchis R (2005). Evolving Consensus in Portal Hypertension Report of the Baveno IV Consensus Workshop on methodology of diagnosis and therapy in portal hypertension. *Journal of Hepatology*.

[14] Bendtsen F, Skovgard LT, Soerensen TIA (1990). Agreement among multiple observers on endoscopic diagnosis of oesophageal varices before bleeding. *Hepatology*.

[15] Cales P, Pascal JP (1990). Gastroesophageal endoscopic features in cirrhosis: comparison of intracenter and intercenter observer variability. *Gastroenterology*.

[16] Winkfield B, Aubé C, Burtin P, Calès P (2003). Inter-observer and intra-observer variability in hepatology. *European Journal of Gastroenterology and Hepatology*.

[17] Garcia-Tsao G, Escorsell A, Zakko M (1997). Predicting the presence of significant portal hypertension and varices in compensated cirrhotic patients. *Hepatology*.

[18] Berzigotti A, Gilabert R, Abraldes JG (2008). Noninvasive prediction of clinically significant portal hypertension and esophageal varices in patients with compensated liver cirrhosis. *American Journal of Gastroenterology*.

[19] Pilette C, Oberti F, Aubé C (1999). Non-invasive diagnosis of esophageal varices in chronic liver diseases. *Journal of Hepatology*.

[20] Chalasani N, Imperiale TF, Ismail A (1999). Predictors of large esophageal varices in patients with cirrhosis. *American Journal of Gastroenterology*.

[21] Zaman A, Hapke R, Flora K, Rosen HR, Benner K (1999). Factors predicting the presence of esophageal or gastric varices in patients with advanced liver disease. *American Journal of Gastroenterology*.

[22] Rockey DC, Bissell DM (2006). Noninvasive measures of liver fibrosis. *Hepatology*.

[23] Galal GM, Amin NF, Abdel Hafeez HA, El-Baz MAH (2011). Can serum fibrosis markers predict medium/large oesophageal varices in patients with liver cirrhosis?. *Arab Journal of Gastroenterology*.

[24] Körner T, Kropf J, Gressner AM (1996). Serum laminin and hyaluronan in liver cirrhosis: markers of progression with high prognostic value. *Journal of Hepatology*.

[25] Bahr MJ, Böker KHW, Horn W, Günzler V, Manns MP (1997). Serum laminim P1 levels do not reflect critically elevated portal pressure in patients with liver cirrhosis. *Hepato-Gastroenterology*.

[26] Gressner AM, Tittor W, Kropf J (1988). Evaluation of serum aminoterminal procollagen type III propeptide as an index of portal hypertension and esophageal varices in chronic liver diseases. *Clinica Chimica Acta*.

[27] Mamori S, Searashi Y, Matsushima M (2008). Serum type IV collagen level is predictive for esophageal varices in patients with severe alcoholic disease. *World Journal of Gastroenterology*.

[28] Imbert-Bismut F, Ratziu V, Pieroni L, Charlotte F, Benhamou Y, Poynard T (2001). Biochemical markers of liver fibrosis in patients with hepatitis C virus infection: a prospective study. *Lancet*.

[29] Poynard T, Morra R, Halfon P (2007). Meta-analyses of FibroTest diagnostic value in chronic liver disease. *BMC Gastroenterology*.

[30] Naveau S, Raynard B, Ratziu V (2005). Biomarkers for the prediction of liver fibrosis in patients with chronic alcoholic liver disease. *Clinical Gastroenterology and Hepatology*.

[31] Thabut D, Naveau S, Charlotte F (2006). The diagnostic value of biomarkers (AshTest) for the prediction of alcoholic steato-hepatitis in patients with chronic alcoholic liver disease. *Journal of Hepatology*.

[32] Thabut D, Trabut JB, Massard J (2006). Non-invasive diagnosis of large oesophageal varices with FibroTest in patients with cirrhosis: a preliminary retrospective study. *Liver International*.

[33] Qamar AA, Grace ND, Groszmann RJ (2008). Platelet count is not a predictor of the presence or development of gastroesophageal varices in cirrhosis. *Hepatology*.

[34] El-Sherif AM, Abou-Shady MA, Al-Bahrawy AM, Bakr RM, Hosny AMM (2008). Nitric oxide levels in chronic liver disease patients with and without oesophageal varices. *Hepatology International*.

[35] Makhlouf MM, Awad A, Zakhari MM, Fouad M, Saleh WA (2002). Vascular endothelial growth factor level in chronic liver diseases. *Journal of the Egyptian Society of Parasitology*.

[36] Tarantino G, Citro V, Esposit P (2009). Blood ammonia levels in liver cirrhosis: a clue for the presence of portosystemic collateral veins. *BMC Gastroenterology*.

[37] Giannini E, Botta F, Borro P (2003). Platelet count/spleen diameter ratio: proposal and validation of a non-invasive parameter to predict the presence of oesophageal varices in patients with liver cirrhosis. *Gut*.

[38] Giannini EG, Zaman A, Kreil A (2006). Platelet count/spleen diameter ratio for the noninvasive diagnosis of esophageal varices: results of a multicenter, prospective, validation study. *American Journal of Gastroenterology*.

[39] Baig WW, Nagaraja MV, Varma M, Prabhu R (2008). Platelet count to spleen diameter ratio for the diagnosis of esophageal varices: is it feasible?. *Canadian Journal of Gastroenterology*.

[40] Agha A, Anwar E, Bashir K, Savarino V, Giannini EG (2009). External validation of the pPlatelet count/spleen diameter ratio for the diagnosis of esophageal varices in hepatitis C virus-related cirrhosis. *Digestive Diseases and Sciences*.

[41] Barrera F, Riquelme A, Soza A (2009). Platelet count/spleen diameter ratio for non-invasive prediction of high risk esophageal varices in cirrhotic patients. *Annals of Hepatology*.

[42] Schwarzenberger E, Meyer T, Golla V, Sahdala NP, Min AD (2010). Utilization of platelet count spleen diameter ratio in predicting the presence of esophageal varices in patients with cirrhosis. *Journal of Clinical Gastroenterology*.

[43] Barikbin R, Hekmatnia A, Omidifar N, Farghadani M, Adibi P (2010). Prediction severity of esophageal varices: a new cutoff point for Platelet count/spleen diameter ratio. *Minerva Gastroenterologica e Dietologica*.

[44] Burton JR, Liangpunsakul S, Lapidus J, Giannini E, Chalasani N, Zaman A (2007). Validation of a multivariate model predicting presence and size of varices. *Journal of Clinical Gastroenterology*.

[45] Nyblom H, Björnsson E, Simrén M, Aldenborg F, Almer S, Olsson R (2006). The AST/ALT ratio as an indicator of cirrhosis in patients with PBC. *Liver International*.

[46] Treeprasertsuk S, Kowdley KV, Luketic VAC (2010). The predictors of the presence of varices in patients with primary sclerosing cholangitis. *Hepatology*.

[47] Castéra L, Bail BL, Roudot-Thoraval F (2009). Early detection in routine clinical practice of cirrhosis and oesophageal varices in chronic hepatitis C: comparison of transient elastography (FibroScan) with standard laboratory tests and non-invasive scores. *Journal of Hepatology*.

[48] Alempijevic T, Bulat V, Djuranovic S (2007). Right liver lobe/albumin ratio: contribution to non-invasive assessment of portal hypertension. *World Journal of Gastroenterology*.

[49] Fraquelli M, Rigamonti C, Casazza G (2007). Reproducibility of transient elastography in the evaluation of liver fibrosis in patients with chronic liver disease. *Gut*.

[50] Roulot D, Czernichow S, Le Clésiau H, Costes JL, Vergnaud AC, Beaugrand M (2008). Liver stiffness values in apparently healthy subjects: influence of gender and metabolic syndrome. *Journal of Hepatology*.

[51] Coco B, Oliveri F, Maina AM (2007). Transient elastography: a new surrogate marker of liver fibrosis influenced by major changes of transaminases. *Journal of Viral Hepatitis*.

[52] Arena U, Vizzutti F, Corti G (2008). Acute viral hepatitis increases liver stiffness values measured by transient elastography. *Hepatology*.

[53] Sagir A, Erhardt A, Schmitt M, Häussinger D (2008). Transient elastography is unreliable for detection of cirrhosis in patients with acute liver damage. *Hepatology*.

[54] Kazemi F, Kettaneh A, N’kontchou G (2006). Liver stiffness measurement selects patients with cirrhosis at risk of bearing large oesophageal varices. *Journal of Hepatology*.

[55] Vizzutti F, Arena U, Romanelli RG (2007). Liver stiffness measurement predicts severe portal hypertension in patients with HCV-related cirrhosis. *Hepatology*.

[56] Bureau C, Metivier S, Peron JM (2008). Transient elastography accurately predicts presence of significant portal hypertension in patients with chronic liver disease. *Alimentary Pharmacology and Therapeutics*.

[57] Jung HS, Kim YS, Kwon OS (2008). Usefulness of liver stiffness measurement for predicting the presence of esophageal varices in patients with liver cirrhosis. *The Korean Journal of Hepatology*.

[58] Stefanescu H, Grigorescu M, Lupsor M, Procopet B, Maniu A, Badea R (2011). Spleen stiffness measurement using fibroscan for the noninvasive assessment of esophageal varices in liver cirrhosis patients. *Journal of Gastroenterology and Hepatology*.

[59] Talwalkar JA, Yin M, Venkatesh S (2009). Feasibility of in vivo MR elastographic splenic stiffness measurements in the assessment of portal hypertension. *American Journal of Roentgenology*.

[60] Vilgrain V (2001). Ultrasound of diffuse liver disease and portal hypertension. *European Radiology*.

[61] Schepis F, Cammà C, Niceforo D (2001). Which patients with cirrhosis should undergo endoscopic screening for esophageal varices detection?. *Hepatology*.

[62] Fleig WE (2001). To scope or not to scope: still a question. *Hepatology*.

[63] Riggio O, Angeloni S, Nicolini G, Merli M (2002). Endoscopic screening for esophageal varices in cirrhotic patients. *Hepatology*.

[64] Perri RE, Chiorean MV, Fidler JL (2008). A prospective evaluation of computerized tomographic (CT) scanning as a screening modality for esophageal varices. *Hepatology*.

[65] Kim SH, Kim YJ, Lee JM (2007). Esophageal varices in patients with cirrhosis: multidetector CT esophagography—comparison with endoscopy. *Radiology*.

[66] Kim YJ, Raman SS, Yu NC, To’o KJ, Jutabha R, Lu DSK (2007). Esophageal varices in cirrhotic patients: evaluation with liver CT. *American Journal of Roentgenology*.

[67] Spinzi G, Terreni N, Mandelli G, Paggi S (2008). Comment on a prospective evaluation of computerized tomographic (CT) scanning as a screening modality for esophageal varices. *Hepatology*.

[68] De Franchis R, Eisen GM, Laine L (2008). Esophageal capsule endoscopy for screening and surveillance of esophageal varices in patients with portal hypertension. *Hepatology*.

[69] Frenette CT, Kuldau JG, Hillebrand DJ, Lane J, Pockros PJ (2008). Comparison of esophageal capsule endoscopy and esophagogastroduodenoscopy for diagnosis of esophageal varices. *World Journal of Gastroenterology*.

[70] Eisen GM, Eliakim R, Zaman A (2006). The accuracy of PillCam ESO capsule endoscopy versus conventional upper endoscopy for the diagnosis of esophageal varices: a prospective three-center pilot study. *Endoscopy*.

[71] Pena LR, Cox T, Koch AG, Bosch A (2008). Study comparing oesophageal capsule endoscopy versus EGD in the detection of varices. *Digestive and Liver Disease*.

[72] Lapalus MG, Soussan EB, Gaudric M (2009). Esophageal capsule endoscopy vs. EGD for the evaluation of portal hypertension: a French prospective multicenter comparative study. *American Journal of Gastroenterology*.

[73] Schreibman I, Meitz K, Kunselman AR, Downey M, Le T, Riley T (2010). Defining the Threshold: new data on the ability of capsule endoscopy to discriminate the size of esophageal varices. *Digestive Diseases and Sciences*.

[74] Lapalus MG, Dumortier J, Fumex F (2006). Esophageal capsule endoscopy versus esophagogastroduodenoscopy for evaluating portal hypertension: a prospective comparative study of performance and tolerance. *Endoscopy*.

[75] Lu Y, Gao R, Liao Z, Hu LH, Li ZS (2009). Meta-analysis of capsule endoscopy in patients diagnosed or suspected with esophageal varices. *World Journal of Gastroenterology*.

[76] Guturu P, Sagi SV, Ahn D, Jaganmohan S, Kuo Y-F, Sood GK (2011). Capsule endoscopy with PILLCAM ESO for detecting esophageal varices: a meta-analysis. *Minerva Gastroenterologica e Dietologica*.

[77] Ramirez FC, Hakim S, Tharalson EM, Shaukat MS, Akins R (2005). Feasibility and safety of string wireless capsule endoscopy in the diagnosis of esophageal varices. *American Journal of Gastroenterology*.

[78] White CM, Kilgore ML (2009). PillCam ESO versus esophagogastroduodenoscopy in esophageal variceal screening: a decision analysis. *Journal of Clinical Gastroenterology*.

[79] Spiegel BMR, Esrailian E, Eisen G (2007). The budget impact of endoscopic screening for esophageal varices in cirrhosis. *Gastrointestinal Endoscopy*.

[80] Kowalski HJ, Abelmann WH (1953). The cardiac output at rest in Laennec’s cirrhosis. *The Journal of clinical investigation*.

[81] Meng HC, Lin HC, Tsai YT (1994). Relationships between the severity of cirrhosis and haemodynamic values in patients with cirrhosis. *Journal of Gastroenterology and Hepatology*.

[82] Moller S, Hobolth L, Winkler C, Bendtsen F, Christensen E (2011). Determinants of the hyperdynamic circulation and central hypovolaemia in cirrhosis. *Gut*.

[83] Kobayashi A, Katsuta Y, Aramaki T, Okumura H (1991). Interrelation between esophageal varices, and systemic and hepatic hemodynamics in male patients with compensated cirrhosis. *Japanese Journal of Medicine*.

[84] Chikamori F, Inoue A, Okamoto H, Kuniyoshi N, Kawashima T, Takase Y (2011). Relationships between types of esophagogastric varices and systemic hemodynamics in patients with liver cirrhosis. *Hepato-Gastroenterology*.

[85] Imholz BPM, Wieling W, Van Montfrans GA, Wesseling KH (1998). Fifteen years experience with finger arterial pressure monitoring: assessment of the technology. *Cardiovascular Research*.

[86] Poon TCW, Hui AY, Chan HLY (2005). Prediction of liver fibrosis and cirrhosis in chronic hepatitis B infection by serum proteomic fingerprinting: a pilot study. *Clinical Chemistry*.

[87] Morra R, Munteanu M, Bedossa P (2007). Diagnostic value of serum protein profiling by SELDI-TOF ProteinChip compared with a biochemical marker, FibroTest, for the diagnosis of advanced fibrosis in patients with chronic hepatitis C. *Alimentary Pharmacology and Therapeutics*.

[88] Gobel T, Vorderwulbecke S, Hauck K (2006). New multiprotein patterns differentiate liver fibrosis stages and hepatocellular carcinoma in chronic hepatitis C serum samples. *World Journal of Gastroenterology*.

[89] Goulis J, Patch D, Burroughs AK (1999). Bacterial infection in the pathogenesis of variceal bleeding. *Lancet*.

[90] Graupera M, GarcíaPagán J, Titos E (2002). 5-Lipoxygenase inhibition reduces intrahepatic vascular resistance of cirrhotic rat livers: a possible role of cysteinyl-leukotrienes. *Gastroenterology*.

[91] Steib CJ, Gerbes AL, Bystron M (2007). Kupffer cell activation in normal and fibrotic livers increases portal pressure via thromboxane A2. *Journal of Hepatology*.

[92] Yokoyama Y, Xu H, Kresge N (2003). Role of thromboxane A2 inearly BDL-induced portal hypertension. *American Journal of Physiology, Gastrointestinal and Liver Physiology*.

[93] Holland-Fischer P, Gronbaek H, Sandahl TD (2011). Kuppffer cells are activated in cirrhotic portal hypertension and not normalised by TIPS. *Gut*.

